# Mental Health for All: The Case for Investing in Digital Mental Health to Improve Global Outcomes, Access, and Innovation in Low-Resource Settings

**DOI:** 10.3390/jcm12216735

**Published:** 2023-10-25

**Authors:** Manuel Faria, Stella Tan Pei Zin, Roman Chestnov, Anne Marie Novak, Shahar Lev-Ari, Michael Snyder

**Affiliations:** 1 Department of Genetics, Stanford University School of Medicine, Stanford, CA 94305, USA; slevari@stanford.edu; 2 Department of Psychology, Stanford University, Stanford, CA 94305, USA; 3 Health and Development, United Nations Development Programme, 1219 Geneva, Switzerland; stella.tan.pei.zin@undp.org (S.T.P.Z.); roman.chestnov@undp.org (R.C.); 4 Department of Health Promotion, Tel Aviv University School of Medicine, Tel Aviv 6997801, Israel; annemarie@mail.tau.ac.il

**Keywords:** digital mental health, mobile health, medical innovation, mental health intervention, telemedicine

## Abstract

Mental health disorders are an increasing global public health concern that contribute to morbidity, mortality, disability, and healthcare costs across the world. Biomedical and psychological research has come a long way in identifying the importance of mental health and its impact on behavioral risk factors, physiological health, and overall quality of life. Despite this, access to psychological and psychiatric services remains widely unavailable and is a challenge for many healthcare systems, particularly those in developing countries. This review article highlights the strengths and opportunities brought forward by digital mental health in narrowing this divide. Further, it points to the economic and societal benefits of effectively managing mental illness, making a case for investing resources into mental healthcare as a larger priority for large non-governmental organizations and individual nations across the globe.

## 1. Introduction

The World Health Organization (WHO) estimates that depression and anxiety disorders cost the global economy a total of USD 1 trillion in lost productivity [1]. Depression is currently the single largest contributor to global disability [2]. Although highly underreported, it was estimated that 970 million people had a mental disorder in 2017, most commonly anxiety or depression [3]. A WHO scientific brief most recently reported a 25% increase in the global rate of mental illness since the onset of the COVID-19 pandemic [4]—a heightened disease burden that cannot be addressed with the average 2% of the total health budget that governments allocate to mental health [5]. In an interview, WHO Director of Mental Health and Substance Abuse, Dévora Kestel, concluded that “[w]hile the pandemic has generated interest in and concern for mental health, it has also revealed historical under-investment in mental health services. Countries must act urgently to ensure that mental health support is available to all” [6]. The WHO brief also noted that certain underserved groups, particularly women and younger people, were the most severely impacted.

One of the biggest challenges of mental health services is access. Some of the leading reasons for the lack of access to mental health services include high costs, provider unavailability or shortage, long distances, lack of time, societal stigma, and many others [7,8]. To address this gap, a myriad of Digital Mental Health Intervention (DMHI) services has been developed over the last 25 years [9]. DMHIs include a diverse set of tools including videoconference-based tele-psychiatry and tele-therapy services, asynchronous self-help tools, and mental health monitoring platforms. These strategies fall under the umbrella of Mobile Health, or mHealth for short, which includes a number of technologically enabled healthcare services that have been recognized by the WHO for their ability to improve the quality and coverage of care [10]. Due to the COVID-19 pandemic, mental health policy changes have already followed suit with advances in mental health to allow for more accessible and safe digital mental healthcare, a novel development that might particularly benefit previously underserved populations [11]. Still, the overwhelming majority of people around the world do not have access to these innovative services despite global internet access standing at 60% [12].

In 2012, the World Health Organization (WHO) and the International Telecommunication Union (ITU) launched the Be He@lthy, Be Mobile (BHBM) initiative to provide support for governments wishing to implement scalable mHealth infrastructure for non-communicable diseases [13]. The initiative provides evidence-based, technology-agnostic resources to upscale digital health services, and currently includes toolkits for chronic illness (e.g., mDiabetes, mHypertension), aging demographics (e.g., mAgeing), and even tobacco use (e.g., mTB, mTobaccoCessation), in addition to some wellbeing apps (e.g., mYoga). While these advancements have been critical, no international DMHI platform or toolkit is available for countries wishing to curtail broader mental illness, nor is there a scholarly analysis to advocate for the investment in such mHealth services despite the WHO’s endorsement thereof [14]. This qualitative literature review explores the costs and benefits of investing in creating a digital mental health treatment infrastructure, particularly in low-resource settings, in order to address this significant global gap.

## 2. Methodology

The present non-systematic review aims to provide a qualitative overview of different scopes of work around the field of digital mental health interventions, as well as their effectiveness and limitations, aiming to assemble a comprehensive and bird’s-eye view of the literature and path towards implementation. The review examined published research articles and public health reports between the dates of July and September 2023. Only English articles were considered. Critical themes and gaps were identified, and a further review was conducted. Articles included both experimental trials (e.g., randomized controlled trials) and observational studies (e.g., longitudinal studies), as well as further incorporation of systematic and meta-reviews when scientific consensus was warranted. DMHIs, for this review, included tele-health; wearable, assistive devices; and other innovations meant to support or treat mental illness. Studies that included or were conducted in low- and middle-income countries were reprioritized.

Lastly, two sample interventions were chosen to highlight the adaptability and efficacy of different digital interventions. Through a comparative analysis of two selected interventions, we aim to underscore the importance of evidence-based approaches in shaping mental health policies and recommendations for the future. We prioritized interventions that were well described and had a substantial impact, thus allowing us to draw meaningful comparisons. Two such interventions were identified for this review: a single-blind, two-arm pragmatic randomized clinical trial among displaced Syrians suffering from depression and impaired functioning in Lebanon, and a pragmatic, multicenter, randomized controlled trial comparing integrated Healthline services with usual care among participants recruited from 43 general practices in 3 areas of England, a high-incidence country. The first study was chosen based on its relevance to addressing mental health disparities among vulnerable populations, and the second due to its potential implications for the broader mental health landscape in a developed country and its practical implementation into an integrated healthcare system.

## 3. Evidence, Efficacy, and Limitations

An essential competency for any healthcare intervention is its efficacy and safety profile, which naturally becomes an ongoing question in the development of mHealth tools. Indeed, large meta-analyses have concluded that mobile phone-based [15] and computerized [16] mental health interventions are both safe and effective [17], albeit with notable heterogeneity. Distinct but similar Digital Behavior Change Interventions have also yielded significant results in promoting healthy behaviors in older adults [18] and children [19] in randomized controlled trials. All together, these findings suggest that technology-enabled services have the potential to be effective at improving psychological and behavioral patterns, and thus warrant the attention of public health authorities for further development, improvement, and implementation.

DMHIs have been empirically studied in multiple mental illnesses, which is an essential feature of their efficacy and disseminability. Digital interventions have been researched for the management of anxiety [20], depression [21], post-traumatic stress disorder [22], substance use disorders [23], eating disorders [24], insomnia [25], obsessive-compulsive symptoms, and suicide [26]. Research has also examined its effect on stress reduction [27], well-being [28], and mindfulness [29], which are key aspects of mental health promotion. Most recently, the role of wearable devices (e.g., smartwatches, fitness trackers) has also been examined as tools that can be integrated into stress management [30] as well as remote diagnoses and monitoring of depression [31,32]. Innovation in biobehavioral tracking stands out as one of the most promising ways to expand healthcare access in settings where appropriate clinical attention may be otherwise impossible. The versatility and customizability of DMHIs point to them as particularly promising when addressing different patient populations, cultural norms, and psychiatric diagnoses.

DMHIs are appealing thanks to their ability to reach remote populations, fit around patients’ schedules, protect anonymity and avoid stigma-related aversion to seeking mental health services, offer on-demand services, and improve affordability. Additionally, DMHIs hold promise for countries with a limited healthcare workforce, especially those with few mental health specialists. DMHIs can offer services by clinical providers (e.g., psychiatrists, psychologists) or non-clinical providers (e.g., counselors, coaches). A meta-analysis of randomized controlled trials found no difference in outcomes between clinical and non-clinical providers utilizing DMHIs [33], suggesting the possibility of upscaling mental health services in countries with a limited number of specialists using DMHIs—a path to healthcare access with the potential to be more time and resource efficient than upscaling the healthcare education pipeline altogether.

**Figure 1:**
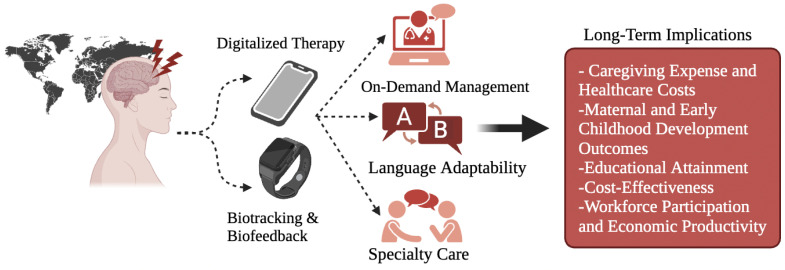
Value generation process for digital and novel interventions in a global context, with short summary of long-term societal and economic implications.

The WHO already offers a guide (i.e., mhGAP-HIG) [34] and a tool (i.e., QualityRights e-Training) to train and certify these non-clinical providers in psychosocial support, the latter of which is already available in 11 languages [35]. A recent randomized controlled trial found that a WHO non-clinically guided digital mental health intervention (i.e., “Step-by-Step”) was clinically effective at reducing depression, anxiety, post-traumatic stress, and functional impairment in displaced Syrian refugees in Lebanon (see Table 1) [36]. Similar results have been found in Western contexts (see Table 2) [37]. This finding is supported by a 2020 meta-analysis suggesting that in low- and middle-income countries (LMICs), which are more severely affected by mental healthcare disparities, DMHIs are still effective at treating mental illnesses, the results being particularly robust for depression and substance use disorders [38]. A previous study estimated that 85–93% of people with anxiety and 79–93% of people with depression are not being treated in LMICs [39]. That same study, however, performed an economic analysis that suggested that increasing treatment for anxiety and depression would cost USD 146 billion yet would roughly yield a USD 310 billion value (i.e., approximately 43 million extra years of healthy life). These intrinsic health benefits are also confluent with an additional USD 399 in net productivity gains. Importantly, the full socio-economic impact of addressing mental health needs has not yet been exhaustively modeled and is therefore possibly underestimated in Chisholm et al.’s analysis (Figure 1 briefly summarizes some implications). The authors point to other, unaccounted variables that may increase (or perhaps decrease) the economic return of mental health treatment. These factors include the effect of maternal depression on early childhood development and educational outcomes, the financial strain of caregiving and unemployment, and the cost of treating physical health conditions for which depression and anxiety are risk factors (e.g., hypertension, stroke, heart disease) [39]. Surely, since LMICs often lack the necessary healthcare infrastructure, workforce, and budget to address their mental health disease burden, they are most likely to greatly benefit from DMHIs that can be remotely distributed, self-administered or non-clinically guided, and affordably scaled. In general, studies repeatedly find the cost-effectiveness of digital interventions [40], but ultimately it is organizations who must decide the fiscal viability of successfully implementation. The prospect of addressing the substantial economic burden of untreated mental illness should be given equal importance to other development strategies, as evidenced by the potential economic gains predicted by the aforementioned model analysis.

However promising, DHMIs have several limitations and challenges to overcome. Some of these challenges include technological literacy, accountability, regulation, smartphone access, and privacy. Additionally, the heterogeneity in outcomes warrants specialized consideration. This variability in outcomes could be attributed to different delivery modes and the individual guidance that patients may need based on their symptoms. While unguided DMHIs seem to be less effective [41], a 2019 meta-analysis found no difference in effectiveness between face-to-face and digitally guided interventions for depression [42]. The mediating factor might be greater patient engagement when guidance is incorporated into treatment, as a 2021 meta-analysis found that more user engagement in DMHIs was associated with better therapeutic efficacy [43]. However, these differences in effectiveness between guided and unguided interventions are likely moderated by symptom severity [21] since more depressed or ill patients are more disengaged as part of their disease process; in fact, the same study found no difference in outcomes in subclinical or mild depression between guided and unguided approaches. In a comprehensive review, Lattie et al. conclude by highlighting the overall robustness of evidence for remote and synchronous as well as web-based DMHIs, while the upcoming data for app- or SMS-based interventions is likewise promising, particularly when paired with human guidance [9]. Altogether, the data support the development, implementation, and further research into digital interventions in real-world clinical settings. Still, less is known about the necessary regulation and certification mechanisms that should exist for clinical and non-clinical providers of these interventions. The WHO, on the flip side, does offer an existing framework of recommendations for digital interventions that take into account both acceptability and harms [44], yet it is ultimately up to national regulatory bodies to decide how to proceed with their own in-country regulation and implementation.

## 4. Innovation and Future Research

The impact of digitalization has profound implications for the long-term growth and sustainability of any healthcare system and its ability to address unmet patient needs. Thus, investing in developing and disseminating DMHIs can have broader implications on the lateral transfer of patient benefits in non-psychiatric sectors. This point is of particular importance for high-need environments, where multiple healthcare priorities may be competing. For instance, researchers have already been able to use wearable sensors (i.e., commercial smart watches or fitness trackers) to predict clinical laboratory results with machine learning algorithms [45]. These technologies function by continuously tracking individual’s vital signs (e.g., body temperature, heart rate, electrodermal activity) in relation to their baseline. Then, via random forest and Lasso models, they may retrospectively predict critical laboratory results only available today through bloodwork [45]. Similar progress has been made to detect psychological stress with wearable devices [46,47], in addition to epileptic seizures [48], accidental falls [49], and cardiovascular abnormalities [50], which are all likewise important unmet healthcare needs. The implications of these findings are quite profound when harmonized with digital interventions. Biowearable tracking may, in the near future, permit the identification of high-risk psychiatric patients before disease onset or severity progression, which even improves DMHI efficacy in this subgroup, and thereby prevents more medically complex interventions. A randomized controlled trial by Crum et al. demonstrated the utility of wearable devises in a mindfulness-based stress management intervention that synchronized wearable tracking with digital biofeedback [30] amongst chronically stressed and anxious employees. As such, digital wearable integration may not only improve the efficacy of current DMHIs but ultimately serve primary and secondary prevention purposes. Although insufficiently researched, the extent of these preventative benefits may extend far beyond mental health and into other chronic disease areas that traditionally hinge on bloodwork for prevention or diagnosis. Yet, the prospective diagnostic ability of wearables is not known. Comparatively, this gives mental health diagnoses the most suitable lunch path with which to explore this nexus further, as they largely rely on clinical questionnaires that already count with prospective validity. Overall, digital wearable integration could be used to address a wide range of medical needs across a person’s lifespan with the same low-cost hardware and be achieved fully remotely. Regarding their ability to address multiple clinical needs, their benefit seems exponential, as innovation improves over time, new applications arise, and economic costs go down.

Another big problem that future innovation may aid in is also preventative and diagnostic in nature. While DMHIs have shown substantial efficacy, there lacks an objective (i.e., non-clinical, non-self-report) measurement of progress. As Jin Jeon et al. point out, this is an active area of research for which digital wearable integration may show promise [31,32]. Authors explain that wearables can quantify a number of diagnostic parameters that have been related or implicated with depression—including gait variability, sleep duration, heart rate variability, electromyography signals, and others—that ultimately bypass limitations such as recall bias, questionnaire inattention, or expectancy effects. Similarly, these are measurement-driven and may permit more personalized DMHIs on the basis of individual symptoms exhibited by patients. Not all psychiatric symptoms seem easily trackable via sensors, however. Therefore, more novel approaches aim to integrate artificial intelligence (AI) with a number of possible data parameters. An example of a promising technology is the use of AI voice biomarkers to diagnose different mental and neurologic illnesses, which has been successful regarding mood and anxiety disorders, as well as cognitive aging conditions [51]. Speech symptom abnormalities have long been associated with psychiatric diseases, and these biomarkers function through machine learning techniques that select linguistic (e.g., speech rate) or acoustic (e.g., pitch entropy) features from which they derive a diagnostic prediction. Voice biomarkers can therefore have the ability to provide non-invasive diagnoses and longitudinal progression tracking within the same digital therapeutic or tele-medicine platforms used by help-seeking patients [51]. This is another innovative feature that may putatively improve the efficacy of DMHIs in real-world settings, especially in places where diagnostic professionals are scarce. Such efficacy trials have not yet been run. Still, the extent to which such voice biomarkers can serve non-Anglophone and non-Western populations is largely unknown, although such a biomarker would be most helpful in non-Anglophone countries. Future research efforts around DMIHs should assess the added value of integrating AI and wearable technologies into existing evidence-based interventions, as the gains from these features may easily scale with little cost in non-Wester countries with limited healthcare access.

## 5. Conclusions

In a world where multiple diseases compete for increased access and prioritization, healthcare systems must evaluate the situational and financial feasibility of large-scale interventions. This review summarizes both the scientific and financial basis for why certain health organizations—especially those in low-resource and/or high-incidence areas—may choose to pursue a digitalized mental health approach as a public health priority. In short, digital mental health interventions enable providers to reach individuals who may not be able to access traditional mental healthcare otherwise. These innovative interventions utilize mobile applications, on-demand platforms, telehealth specialty consults, behavior change protocols, and artificial intelligence technology. These new approaches are both safe and effective in the management of most common psychiatric disorders that tax healthcare systems—disorders that have alarmingly increased since the onset of the COVID-19 pandemic. They are affordable and scalable, which makes them financially tenable as a means to reach high volumes of patients in remote, underserved, or dangerous environments (e.g., warzones). Yet, the treatment of mental illness can have paramount returns over investment: productivity gains, disability-free lifespan, lower long-term healthcare expenditure, and higher labor participation. These economic gains, in return, may serve as a funding source for quality improvement, innovation investment, and other regional healthcare needs. Research developments in this area, especially in biobehavioral wearable tracking and medico-digital integration, are promising prospects that may extend the range and democratization of clinical data that could be used to improve remote diagnosis and clinical monitoring at increasingly lower costs. This review, however, leaves an open question as to the means by which such a perineal technology may be regulated or monitored, urging further research into this matter. Overall, the strengths and promises of digital mental health present an attractive case for investing in these interventions and should be seriously considered amongst healthcare systems and large public health authorities alike.

## Figures and Tables

**Table 1:** Sample Intervention #1, WHO Step-by-Step Intervention [36].

Study	WHO Step-by-Step Intervention
**Country, region**	Lebanon, WHO Eastern Mediterranean region
**Summary**	A single-blind, 2-arm pragmatic randomized clinical trial, comparing guided Step-by-Step with enhanced care as usual (ECAU) among displaced Syrians suffering from depression and impaired functioning in Lebanon
**Conditions**	Depression, impaired functioning
**Intervention**	Step-by-Step is a 5-session intervention, designed to treat depression through an internet-connected device (hybrid app for iOS, Android, and web browsers). It includes 5 story sessions (divided into 3 smaller parts; 20 min taken to read altogether) which are illustrated, and audio recorded. Users are recommended to complete 1 session per week, amounting to a total period of 5 to 8 weeks. It is psychoeducation and training in behavioral activation through an illustrated narrative, with additional therapeutic techniques such as stress management, gratitude exercise, positive self-talk, strengthening social support, and relapse prevention. Users were supported by trained non-specialists (“e-helpers”) who offered weekly phone or message-based contact with users to provide support (up to 15 min per week). Users who accessed the intervention received email or phone notifications.
**Outcome measure**	Primary outcomes: depression (Patient Health Questionnaire, PHQ-9) and impaired functioning (WHO Disability Assessment Schedule-12, WHODAS) at post-treatment. Secondary outcomes: subjective well-being, anxiety, post-traumatic stress, and self-described problems.
**Study size**	A total of 569 participants were included in the study, with 283 randomized to the intervention and 286 to ECAU (enhanced care as usual).
**Impact**	Significant treatment effects were seen for both primary outcomes, depression, and functional impairment. Effect sizes were moderate for both primary outcomes. At 3 months follow-up, the intervention effect was maintained for depression (moderate to large effect sizes) and functional impairment (moderate effect size). For secondary outcomes, significant treatment effects were seen on all outcomes with moderate effect sizes. At 3 months follow-up, the intervention continues to be more significantly effective than ECAU.
**Data collected**	Primary Outcomes: baseline, post-test, and follow-up mean and standard deviation of scores for PHQ-9 and WHODAS. Secondary Outcomes: baseline, post-test, and follow-up mean and standard deviation of scores for WHO-5, GAD-7, PCL-5, and PSYCLOPS.
**Fit-for-purpose**	Relevance: the study addresses the pressing need for accessible mental health care for displaced populations, particularly in low- and middle-income countries where such services are scarce. Completeness: the study design is comprehensive, employing a single-blind, 2-arm pragmatic randomized clinical trial and a good sample size (*n* = 569). The tools used are realistic and forward-thinking. Accuracy: the study followed a well-defined protocol, and the intervention was delivered consistently across participants. Use of validated instruments, intention-to-treat analyses, multiple imputation methods to address missing data, and sensitivity analyses to evaluate bias. Timeliness: the study was conducted during the COVID-19 pandemic and addressed the urgent need for scalable mental health care in crisis-affected regions like Lebanon. Interpretability: clear and comprehensive results, with effects sized for primary and secondary outcomes. The intervention is shown to be effective in reducing mental health problems among displaced Syrians in Lebanon. The study emphasized need for further research.

**Table 2:** Sample Intervention #2, Integrated Healthline Services [37].

Study	The Healthline Services: Integrated Telehealth Service for Patients with Depression
**Country, region**	England, WHO/Europe
**Summary**	A pragmatic, multicenter, randomized controlled trial comparing integrated Healthline Services with usual care among participants recruited from 43 general practices in 3 areas of England.
**Conditions**	Major Depression
**Intervention**	The Healthline Service consisted of regular telephone calls from non-clinical, trained health advisers who followed standardized scripts generated by interactive software. After an initial assessment and goal-setting telephone call, the advisers called each participant on six occasions over 4 months, and then made up to three more calls at intervals of roughly 2 months to provide reinforcement and to detect relapse. Advisers supported participants in the use of online resources (including computerized cognitive behavioral therapy) and sought to encourage healthier lifestyles, optimize medication, and improve treatment adherence. To be eligible, participants needed to have access to the internet and email.
**Outcome measure**	Primary outcome: proportion of participants responding to the intervention at 4 months after start of intervention (achieved PHQ-9 < 10 and reduction in PHQ-9 of ≥5 points). Baseline score of participants was PHQ-9 of at least 10.
**Study size**	A total of 609 participants were recruited, with 307 randomly assigned to Healthline Services plus usual care and 302 to usual care. Final sample size, *n* = 525
**Impact**	The primary outcome showed that 27% of participants in the intervention group responded to treatment at 4 months, compared with 19% of 270 participants in the control group (adjusted odds ratio 1.7, 95% CI 1.1–2.5, *p* = 0.019). There was an overall treatment effect from all follow-up data at 4, 8, and 12 months in two repeated measures analyses, binary and continuous, suggesting a positive average effect of the intervention over the 12-month follow-up period. Mean PHQ-9 scores also decreased over time for both groups. Participants in the intervention group expressed greater satisfaction with access to health care, treatment, and amount of support they received. There was no evidence of improved adherence to antidepressant medication or improved care coordination or that participants made more use of other health-related technologies.
**Data Collected**	Primary outcome: baseline, post-test, and follow-up at 4, 8, and 12 months mean and standard deviation of scores for PHQ-9. Secondary outcome: baseline, post-test and follow-up at 4, 8, and 12 months mean and standard deviation of scores for GAD-7, EQ-5D-5L, heiQ, Morisky, eHEALS, and Haggerty.
**Fit-for-Purpose**	Relevance: the study design and intervention were relevant to address the need for expanding mental health care and improving outcomes for patients with depression. Completeness: the intervention was comprehensive and studied, addressing various components of the TECH model, which included realistic telehealth tools with evidence of effectiveness for depression. It included a good sample size of *n* = 525. Accuracy: the study followed a well-defined protocol, and the intervention was delivered consistently across participants. It used validated instruments, multiple imputation methods to address missing data, repeated measures analyses, mixes-effects logistic regressions, and sensitivity analyses. The study reported adjusted odds rations with confidence intervals as well as effect sizes for the secondary outcomes. Timeliness: the study was published recently and addressed ongoing challenges in mental health service delivery, at a time when mental health issues continue to be significant public health concerns worldwide. Interpretability: clear and comprehensive results, showing the intervention to be effective as a mental health care tool for patients with depression. The study highlighted the challenges in achieving sustained engagement with the intervention.

## Data Availability

No new data were created or analyzed in this study. Data sharing is not applicable to this article.
